# Review of the Anti-*Candida albicans* Activity and Physical Properties of Soft Lining Materials Modified with Polyene Antibiotics, Azole Drugs, and Chlorohexidine Salts

**DOI:** 10.3390/ma17215383

**Published:** 2024-11-04

**Authors:** Izabela Barszczewska-Rybarek, Patrycja Kula, Grzegorz Chladek

**Affiliations:** 1 Department of Physical Chemistry and Technology of Polymers, Faculty of Chemistry, Silesian University of Technology, Strzody 9 Str., 44-100 Gliwice, Poland; patrycja.kula@polsl.pl; 2 Materials Research Laboratory, Faculty of Mechanical Engineering, Silesian University of Technology, Konarskiego 18A Str., 44-100 Gliwice, Poland; grzegorz.chladek@polsl.pl

**Keywords:** soft lining materials, antifungal properties, *Candida albicans*, polyene antibiotics, azole drugs, chlorhexidine salts, mechanical properties

## Abstract

This review examined the current state of knowledge on the modifications of commercial soft lining materials (SLMs) with a variety of antifungal compounds: (i) polyene antibiotics, including nystatin and amphotericin B, (ii) azole drugs, including fluconazole, itraconazole, clotrimazole, ketoconazole, and miconazole, and (iii) antiseptics, including chlorhexidine salts to give them anti-*Candida albicans* properties. The effect of such modifications on the SLMs’ physical properties, such as drug release, water sorption, surface properties, bond strength, tensile strength, and hardness, was also analyzed. In effect, this study provided a unique compilation of research results obtained for numerous properties of SLM modified with antifungal compounds that differ in their chemical structure and mechanism of antifungal action. These results might also be useful for prosthetic dentistry, where SLMs are used to prevent and treat candidiasis, the most common disease among denture wearers.

## 1. Introduction

The WHO Global Oral Health Report of 2022 revealed that nearly 3.5 billion people worldwide are affected by oral diseases, primarily caries and periodontal disease. Severe and long-term dental caries lead to tooth loss, which affects almost 7% of the world’s population aged 20 to 60 and 23% of people over 60 [1].

Removable dentures offer a practical solution for those experiencing partial or complete tooth loss. However, denture wearers often encounter soft tissue abrasions and pain. It usually affects patients with thin, atrophic ridges, bony undercuts, inelastic mucosa, bruxism, and xerostomia [2,3]. Discomfort while wearing dentures results from stresses caused by chewing forces on the gums, especially over the areas supporting the dentures [4,5]. Applying a soft lining material (SLM) to the surface of a rigid denture base that comes into contact with the underlying tissues can offer even stress distribution, promote healing of irritated tissues, and provide a chance to relieve the pain and discomfort of denture wearers [6,7]. These materials have a shock-absorbing effect, acting as a soft cushion separating the hard denture plate from the delicate tissues of the oral cavity. Further advantages of SLMs include biocompatibility, low cytotoxicity, improvement of denture positioning in the oral cavity, and good mechanical properties, including low hardness, high elasticity, and high bond strength between the SLM and the denture [5,8,9,10]. These materials are recommended for patients experiencing pain or discomfort associated with wearing dental prostheses and during recovery after surgical or medical procedures [11].

Although SLMs have numerous advantages, they do not prevent candidiasis, a disease caused by *C. albicans*. It is the most common fungal infection in denture wearers, which can significantly reduce the quality of life of patients. If left untreated, the infection can spread throughout the body and cause diseases in organs such as the kidneys, heart, lungs, and liver [12,13,14,15,16,17,18,19,20].

*C. albicans* is the largest biofilm producer among *Candida* spp. [15,21,22,23,24,25,26,27] and is part of the normal flora of the digestive tract in 40 to 80% of the human population. Other *Candida* spp., such as *Candida parapsilosis*, *Candida dubliniensis*, *Candida tropicalis*, *Candida krusei*, and *Candida glabrata*, are also commonly present in the oral cavity of healthy individuals [28,29].

Candidiasis occurs when the epithelium covering the gums or oral mucosa is damaged, usually by the dentures rubbing against the oral tissues, causing recurring injuries [25,30,31,32]. The risk of candidiasis is higher in the immuno-compromised or elderly patients [33].

The prevention and treatment of candidiasis involves maintaining oral hygiene, cleaning and disinfecting dentures, replacing old dentures, not wearing dentures at night, changing eating habits, and using topical and systemic antifungal treatments [34]. In some cases, the reduction of microbial colonies is insufficient [35,36]. To prevent candidiasis and its recurrence, it is best to minimize the contact between the denture plate and the soft tissue of the oral cavity by applying the SLM on the denture surface [37,38,39]. However, commercially available materials do not have antifungal activity [40] and may even promote *C. albicans* colonization [39,41]. This explains why scientists are working on developing SLMs with antifungal properties.

The success of using antifungal medicines and antiseptics to treat candidiasis led to the idea of incorporating them into SLMs, which then became controlled drug delivery systems. As the antifungal compound is gradually released from the material, it would create an antifungal effect between the SLM surface and the oral mucosa.

Despite the beneficial aspects of modifying SLMs with antifungal medicines and antiseptics for therapeutic purposes, it can compromise their physico-mechanical properties [42,43,44,45].

There is limited literature that summarizes the findings of studies on SLMs with antifungal properties. In 2016, Iqbal et al. reviewed the literature on stomatitis treatment with selected antifungal agents [46]. In 2021, Shaikh et al. summarized the antifungal properties of SLMs modified with nystatin [47]. Finally, in 2022, Yudaev et al. focused on inorganic antifungal agents in various polymeric dental materials [48].

This review offers a comprehensive discussion on the influence of modifications of commercially available SLMs with medicines and antiseptics on various properties of those products. For the first time, the results achieved for polyene antibiotics (nystatin and amphotericin B), azole drugs (fluconazole, itraconazole, ketoconazole, miconazole, and clotrimazole), and antiseptics (chlorhexidine salts) were summarized in one review article. Additionally, this review gathers results for all studied properties to date, including their cytotoxicity, antifungal, antibacterial, physical, and mechanical properties.

## 2. Classification of SLMs

The ISO standards ISO 10139-1 “Dentistry—Soft lining materials for removable dentures—Part 1: Material for short-term use” [49] and ISO 10139-2 “Dentistry—Soft lining materials for removable dentures—Part 2: Material for long-term use” [50] regulate many issues related to the SLMs. However, the classification of these materials is not consistent. They are categorized by the use time, chemical composition, and gelation mechanism.

**Figure 1:**
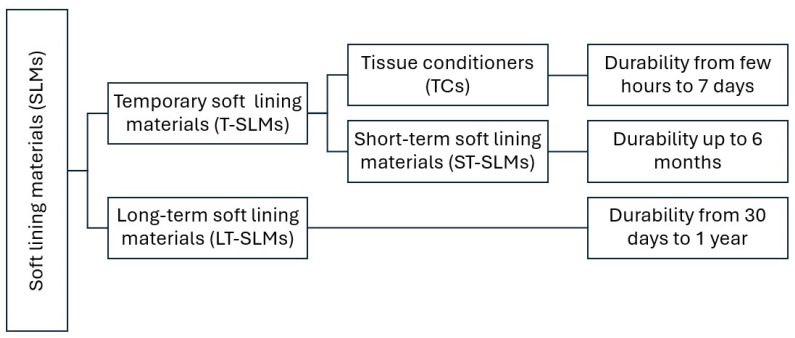
Classification of SLMs according to the use time.

According to the use time, temporary soft lining materials (T-SLMs) and long-term soft lining materials (LT-SLMs) can be distinguished. T-SLMs include tissue conditioners (TCs) and short-term soft lining materials (ST-SLMs) (Figure 1) [49,50,51,52,53].

The use time is closely related to the durability, which, depending on the material, can range from several hours to one year. SLMs that retain stable properties from a few hours to half a year after the application are temporary materials. They include TCs and ST-SLMs. Those that maintain durability up to one year after the application are LT-SLMs [2].

SLMs can be categorized by the chemical composition into acrylic soft lining materials (A-SLMs), and silicone soft lining materials (S-SLMs). Additionally, there are other less common materials comprising polyurethanes, vinyl copolymers, and polyphosphazenes [49,50,51,52,53].

Another classification of SLMs was based on their gelation mechanism. Some undergo a sol–gel transition as a result of plasticization, which is a physical process. Others are subjected to both plasticization and polymerization, the latter being a chemical reaction [2,54,55,56].

TCs solidify due to the plasticization of a linear methacrylate polymer at room temperature. Therefore, they are also called self-curing A-SLMs. They consist of a powder and liquid, in which the powder component is usually poly(ethyl methacrylate) (PEMA) with molecular weight from 1.79 × 10^5^ and 3.25 × 10^5^ [57], whereas the liquid component is a mixture of a plasticizer—most often an aromatic ester and a solvent—usually ethanol [58,59,60]. The plasticizer and solvent give the SLMs an appropriately low hardness and high elasticity [56]. However, their molecules have a tendency to wash out in saliva, and TCs quickly lose shock-absorbing properties (elasticity decreases and hardness increases). Therefore, their recommended lifetime is the shortest among SLMs and ranges from several hours to seven days [55]. Manufacturers refer to TCs as temporary relines on safety data sheets. Commercial TCs, their chemical compositions, component ratios, and literature references related to their modifications for antifungal properties are listed in Table 1.

ST-SLMs are of the A-SLM type and consist of powder and liquid. Their powder component is made up of a polymer—PEMA or poly(methyl methacrylate (PMMA) and the initiator of the free radical polymerization—benzoyl peroxide (BPO) [55,57,82]. The liquid component is a mixture of methacrylate monomers, such as methyl methacrylate (MMA), ethyl methacrylate (EMA), n-butyl methacrylate (BuMA), ethylene glycol dimethacrylate (EGDMA), triethylene glycol dimethacrylate (TEGDMA), a solvent—usually ethanol or ethyl acetate, a plasticizer—usually aromatic ester, and an accelerator of free radical polymerization (reducer in a redox-type initiating system), which is most often *N,N*-dimethyl-*p*-toluidine (DMPT) [83]. They solidify by simultaneous plasticization and polymerization at increased temperatures (so-called heat-curing A-SLMs) [55]. Monomethacrylates, such as MMA, EMA, and BuMA, are bifunctional monomers and form linear polymers. Dimethacrylates, such as EGDMA TEGDMA, are tetrafunctional monomers, and therefore, their presence in the polymerizing systems results in crosslink polymers [84]. The coherent polymer matrix formed throughout the gelation process, particularly crosslinked, limits the leaching of plasticizer and solvent into saliva. As a result, the durability of ST-SLMs exceeds that of TCs. Consequently, their suggested lifetime is longer, specifically up to six months [55,72]. Commercial ST-SLM, its chemical compositions, component ratio, and literature reference related to its modifications for antifungal properties is shown in Table 2.

LT-SLMs are typically S-SLMs, which can be one- or two-component systems. Single-component S-SLMs consist of vinyl-terminated polydimethylsiloxanes, organic peroxide (such as benzoyl peroxide), and inorganic fillers. Their polymerization occurs at high temperatures. Two-component S-SLMs consist of catalyst paste, which is a mixture of vinyl-terminated polydimethylsiloxanes and platinum catalyst, and base paste, which is a mixture of vinyl-terminated polydimethylsiloxanes and hydride-terminated polydimethylsiloxanes [55,86,87]. The reaction between the catalyst and the base occurs at room temperature. Both types of S-SLMs result in crosslinked silicone elastomers [88]. These elastomers do not need added plasticizers because they achieve high elasticity and low hardness due to the extremely high elasticity of the siloxane bond [88]. As a result, S-SLMs have long-lasting durability, making them suitable for both short-term and long-term use [55,72]. However, their hydrophobic nature makes them poorly bond to the prosthesis, which is their main drawback [89]. Commercial S-SLMs, their chemical compositions, component ratios, and literature references related to their modifications for antifungal properties are listed in Table 3.

LT-SLMs can also be A-SLMs. However, due to the need to add a plasticizer and solvent, there are not many commercial products of this type. Commercial long-term A-SLMs, their chemical compositions, component ratios, and literature references related to their modifications for antifungal properties are listed in Table 4.

Using an SLM depends on the patient’s age and health condition. TC and ST-SLM are recommended for poorly fitting dentures and for the wound healing of oral tissues [2]. LT-SLMs are recommended for edentulous patients with atrophic alveolar ridges and atrophic mucosa [55,94].

## 3. Antifungal Compounds Used in Clinical Dentistry

### 3.1. Polyene Antibiotics

There are two groups of medicines with antifungal properties: polyene antibiotics and azole derivatives.

The most common polyene antibiotics include nystatin and amphotericin B. Nystatin has the widest spectrum of activity among the available antifungal medicines [39,43,78,95]. It is used for topical treatment of oral thrush and denture stomatitis [96]. Amphotericin B also has antifungal activity, but some amphotericin B-resistant *C. albicans* isolates exist [97]. Since it can cause a number of side effects, it is used to treat serious, life-threatening systemic fungal infections [96]. The minimum inhibitory concentration (MIC) of nystatin and amphotericin B show that both have good antifungal activity against *C. albicans* (Table 5) [97]. Arikan et al. found that MIC for nystatin and amphotericin B-susceptible *C. albicans* isolates was from 0.125 to 1 mg/mL and from 0.125 to 0.5 µg/mL, respectively. However, MIC for nystatin against amphotericin B-resistant *C. albicans* isolates was significantly higher, from 4 to 16 µg/mL [97].

**Figure 2:**
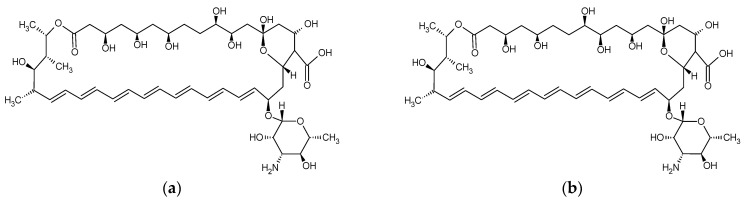
Chemical structure of polyene antibiotics: (**a**) nystatin; (**b**) amphotericin B. Based on [106].

The antifungal action of nystatin and amphotericin B (Figure 2) involves binding to ergosterol, a key component of the fungal cell membrane. It increases the cell membrane permeability, leading to the cell death from the loss of potassium cations and other cytoplasmic components [103].

### 3.2. Azole Drugs

**Figure 3:**
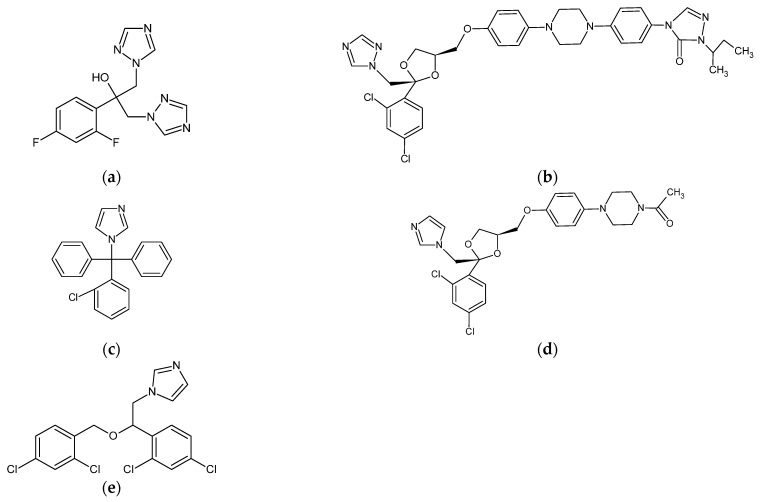
Chemical structure of azole drugs: (**a**) fluconazole; (**b**) itraconazole; (**c**) clotrimazole; (**d**) ketoconazole; and (**e**) miconazole. Based on [106].

Azole drugs are commonly used in the systemic treatment of oral candidal infections [43,95]. They include compounds with triazole rings, such as fluconazole and itraconazole, and compounds with imidazole rings, such as clotrimazole, ketoconazole, and miconazole (Figure 3) [106,107].

The antifungal action of azoles involves interfering with the biosynthesis of ergosterol by inhibiting sterol 14α-demethylase (cytochrome P-450-dependent enzyme). It is responsible for the demethylation of lanosterol and ergosterol formation. Because ergosterol is present in the cell membrane of fungi and not in animal cell membranes, it is a target for azoles. Consequently, the formation of toxic by-products and an increase in the permeability of the fungal cell wall lead to the death of the fungal cell [34,103,106].

The MIC values for azole drugs are presented in Table 6. They show that itraconazole, with the lowest MIC value, had the highest antifungal activity. In addition, fluconazole and itraconazole have higher bioavailability than other azole compounds [92]. Therefore, they can be used in lower doses, making them less toxic [107]. Fluconazole is the most common alternative to nystatin treatment [92,98].

The following is a discussion on the drug concentration in the SLM to achieve an antifungal effect should be based on its recommended daily dose. As drugs have therapeutic effects at different concentrations, some are effective at higher concentrations, and no side effects are observed, whereas others produce toxic effects even at lower doses.

The comparison of the MIC values (Table 5 and Table 6) and recommended daily doses (Table 7) of azole drugs showed that all have a fungistatic effect at concentrations lower than the recommended daily doses.

### 3.3. Chlorohexidine Salts

Chlorhexidine is a well-known antiseptic and disinfectant with low toxicity, antibacterial, and antifungal properties [114,115]. It is widely used in the prevention and topical treatment of oral infections, including candidiasis [20,43,95]. To date, no strains of *C. albicans* resistant to chlorhexidine have been identified, unlike certain strains that showed resistance to antibiotics and azole drugs [116,117].

**Figure 4:**
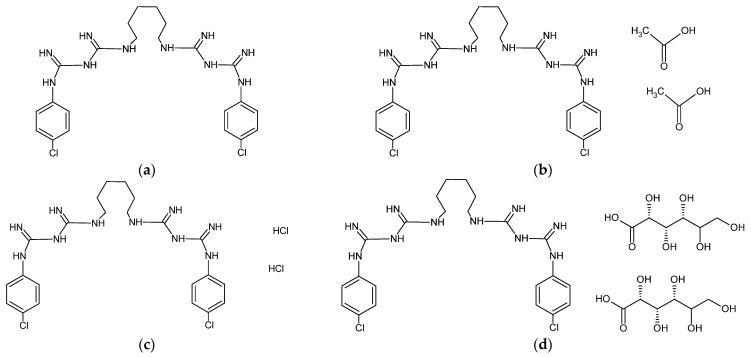
Chemical structure of (**a**) chlorhexidine (based on [119]); (**b**) chlorhexidine diacetate (based on [64]), (**c**) chlorhexidine dihydrochloride (based on [64]); and (**d**) chlorhexidine digluconate (based on [77]).

Chlorhexidine is a cationic guanidine derivative consisting of two (p-chlorophenyl)guanide units linked by a hexamethylene bridge. As it only exists at a pH greater than 12, it is commercially available as diacetate, digluconate, dihydrochloride, and phosphonate salts (Figure 4) [115,118].

The antifungal activity of chlorhexidine salts is explained by various mechanisms, including their adherence to the phospholipids of the fungal cell wall by binding to a glucan fragment, which leads to cell wall damage and the release of potassium ions [91,119,120,121], inhibition of germ tube formation [122], and prevention of the adhesion of blastospores to the epithelial cells and denture surface [120].

The MIC values of the chlorhexidine salts listed in Table 8 show that chlorhexidine diacetate has the highest MIC value, and therefore, it has to be used in the highest concentrations to inhibit the *C. albicans* growth.

## 4. Antifungal Activity

Several studies have examined the antifungal properties of SLMs modified with medicines and antiseptics against *C. albicans* [69,71,77,80,91,93,120,126]. Other strains, such as *C. tropicalis* and *C. krusei*, were also occasionally investigated [28,71].

The methodology for modifying SLMs varied based on their type and the antifungal compounds used. Typically, TCs and A-SLMs were modified by mixing a drug-loaded powder with a liquid component [34,39,62,63,70,71,72,75,76,78,89]. Occasionally, A-SLMs were modified by incorporating a drug in gel form into the mixture of liquid and powder before the gelation process [77]. S-SLMs were typically modified utilizing this second methodology [92,93]. The chlorhexidine salt in a powder form was introduced into the powder component of the SLM [69,120], or a solution of chlorhexidine salt was introduced into the mixture of the powder and liquid components [77,91]. SLMs were also immersed in an aqueous solution of a chlorhexidine salt [80].

### 4.1. Polyene Antibiotics

Douglas et al. started research on the antifungal properties of SLMs and investigated antifungal activity of three commercial TCs, Visco-GEL, Tempo and COE COMFORT against *C. albicans* (Table 1). They found that Tempo exhibited a slight antifungal effect observed for 4 days, while Visco-GEL and COE COMFORT did not show antifungal activity [126]. This could be related to the differences in the ethanol content in SLMs, with Tempo having the highest ethanol concentration, which was 25 wt.% [127]. COE COMFORT and Visco-GEL have significantly lower ethanol content, ranging from 5 to 10 wt.% [128] and from 3 to 10 wt.%, respectively [129]. Even though ethanol is a weak fungicide (the MIC of ethanol against *C. albicans* is 5 to 10 mg/mL) [109], one can hypothesize that the introduction of at least 25 wt.% of ethanol into the SLM can result in its slight antifungal effect. After finding that the neat Visco-GEL was ineffective in inhibiting *C. albicans* growth, Douglas et al. successfully enhanced its fungicidal activity by introducing nystatin into the formula [126].

#### 4.1.1. Nystatin

Thomas et al. demonstrated that the fungicidal effect of the nystatin-loaded SLM depends on the fungal strain, drug concentration, and time. They found that the Visco-GEL TC (Table 1) loaded with nystatin 500,000 and 1,000,000 IU exhibited the fungicidal effect against three fungal species—*C. albicans*, *C. tropicalis*, and *C. krusei*. They showed that the higher the nystatin concentration, the longer the time of the SLM antifungal activity. The 500,000 IU nystatin-loaded Visco-GEL ensured 100% fungicidal effectiveness for 28 days against *C. albicans*, and *C. krusei* and only for 9 days against *C. tropicalis*. After 42 days of the study, the fungicidal effectiveness dropped to 72%, 74%, and 0%, respectively, for *C. albicans*, *C. krusei*, and *C. tropicalis*. The 1,000,000 IU nystatin-loaded Visco-GEL had 100% fungicidal effectiveness for 42 days against *C. albicans* and *C. krusei* and only for 35 days against *C. tropicalis.* After 42 days, the fungicidal effectiveness against *C. tropicalis* dropped to 75%. These findings indicated that the *C. tropicalis* strain is more resistant to nystatin-loaded Visco-GEL compared to the *C. albicans* and *C. krusei* species [71].

**Figure 5:**
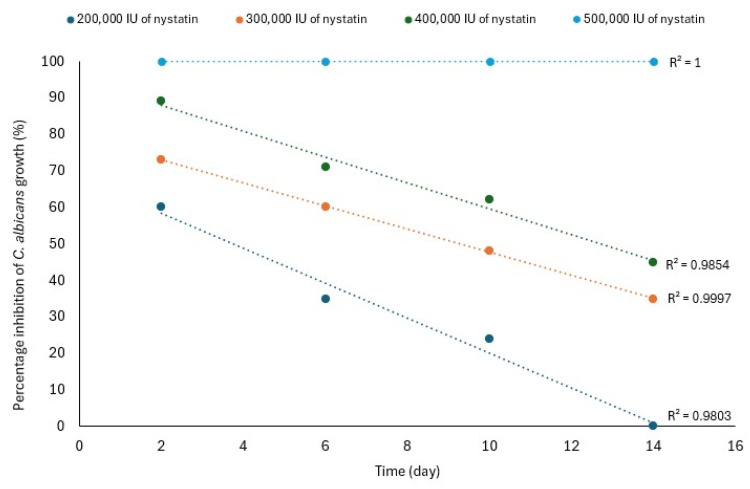
Relationship between the percentage inhibition of *C. albicans* growth for Visco-GEL modified with various concentrations of nystatin, measured over 14 days. Based on [70]).

Sunsil et al. confirmed that the antifungal effect of the nystatin-loaded SLM depends on the drug concentration and time. They loaded Visco-GEL with different amounts of nystatin (200,000, 300,000, 400,000, and 500,000 IU) and determined the percentage inhibition of *C. albicans* growth after 2, 6, 10, and 14 days. A total inhibition of *C. albicans* growth was observed for 14 days with 500,000 IU of nystatin. Lower doses of nystatin did not result in a complete inhibition of fungal growth. For example, only 24% inhibition of *C. albicans* growth was observed for the material with 200,000 IU of nystatin after 10 days. This allowed us to conclude that the minimum effective concentration of nystatin in the SLM is 500,000 IU, which is only 31% of the daily dose of this medicine (taking 1,600,000 IU as its value, Table 7) [81]. Interestingly, a mathematical analysis of the percentage inhibition of *C. albicans* growth and time revealed a linear relationship between these parameters (Figure 5). It can be seen that the longer the SLM’s service time, the lower the percentage inhibition of *C. albicans* growth, except for the material loaded with 500,000 IU of nystatin, for which no time effect was observed for 14 days [70].

Truhlar et al. tested the antifungal activity against *C. albicans* of two TCs, Visco-GEL and Lynal (Table 1), loaded with nystatin 50,000, 100,000, 300,000, 500,000, and 1,000,000 IU over 14 days. They performed the slant agar assay to measure the maximum agar depth of inhibition (MADI) in the aqueous and nonaqueous environments. They found that MADI values for the nystatin-loaded Visco-GEL were higher than those for Lynal, indicating that Visco-GEL was a more efficient nystatin release system. Another finding showed that the antifungal activity decreased over time, with the most significant drop observed after 2 days, and the antifungal activity was higher in the aqueous environment than the nonaqueous environment. Finally, the authors confirmed the 500,000 IU concentration of nystatin as the optimum to achieve a satisfactory antifungal effect of SLMs. This concentration was the minimum required for effective antifungal properties; however, its increase to 1,000,000 IU had no additional impact [75].

Chow et al. confirmed that the fungicidal effect of the nystatin-loaded SLM can depend on the material type, drug concentration, service time, and environment (saliva). In that study, three TCs, Coe-Soft, Visco-GEL, and Fitt (Table 1) were loaded with 1, 3, 5, 7, 9, and 11 wt.% of nystatin and tested for antifungal activity against *C. albicans* (the growth inhibition zone was measured after 5, 17, 20, 23, 41, 44, 65, and 89 h, and then daily for 14 days). The results showed that the nystatin-loaded Fitt material was more effective in inhibiting fungal growth when compared to both Coe-Soft and Visco-GEL. The other observation was that the growth inhibition zone increased as the nystatin concentration increased to 5 wt.% and then slightly decreased, indicating that nystatin 5 wt.% can be recognized as an optimal concentration in SLMs. The largest inhibition zone diameter (IZD) was observed for nystatin 5 wt.% after 65 h. It ranged from 6.3 to 8.7 mm (without saliva) and from 13.7 to 20.0 mm (with saliva). After that time, the fungicidal effect decreased and reached a plateau. It also demonstrated that saliva enhanced the inhibitory effect of the nystatin-loaded SLMs, suggesting increased diffusion in the salivary environment [62].

Barua et al. confirmed antifungal activity against *C. albicans* of Visco-GEL (TC, Table 1) loaded with nystatin 5 and 10 wt.%. They did not observe a significant growth in the IZD with increasing nystatin concentration. The IZD was 31 and 32 mm, respectively, after 24 h and 7 days, regardless of the nystatin concentration [72]. Their findings confirmed that 5 wt.% of nystatin is optimal to achieve a TC with antifungal activity.

Chopde et al. confirmed the antifungal effect against *C. albicans* of the nystatin-loaded Visco-GEL TC (Table 1) in the disk diffusion agar test. The IZD they determined was 10.42 mm. They also investigated the nystatin-loaded GC Soft Line TC (Table 1). They observed the presence of a growth inhibition zone around the samples, with an IZD of 10.5 mm, which was similar to that found for Visco-GEL [39].

Bueno et al. added nystatin 3 wt.% to another two TCs, Softone and Trusoft (Table 1). They showed that the growth of over 90% of *C. albicans* colonies was inhibited for 14 days [34].

Falah-Tafti et al. demonstrated that the Acropars TC (Table 1), when loaded with nystatin at concentrations of 1, 3, 5, and 10 wt.%, completely prevented the colonization of *C. albicans* in each case [78]. It showed that they obtained an optimum antifungal effect at nystatin 1 wt.%, which was a lower concentration than those determined by other authors (3 wt.% [34], 5 wt.% [62,72], 500,000 IU [70,75]). This suggests that antifungal activity may vary depending on the SLM.

Kumpanich et al. showed that the introduction of 20 vol./vol.% nystatin oral suspension into the GC Soft Liner TC (Table 1) resulted in an antifungal activity against *C. albicans*. The inhibition zone appeared around the samples of the modified TC (IZD was 16.59 mm), while it did not appear around the neat TC samples [79].

Songsang et al. showed that the introduction of 30% nystatin oral suspension into three TCs, GC Soft Liner, Visco-GEL, and COE COMFORT (Table 1), resulted in antifungal activity against *C. albicans*, as the inhibition zone was observed around the samples of modified TCs, while it did not appear around the samples of neat TCs. The IZD was 12, 11, and 12 mm, respectively [61].

Hyun-Jin et al. proposed a different approach to the nystatin introduction into SLM. They modified the Dura Conditioner TC (Table 1) by introducing 0.1 wt.% of nystatin-alginate microparticles. The disc diffusion agar test against *C. albicans* revealed the appearance of a growth inhibition zone around the samples of modified material, in contrast to the neat material, where no growth inhibition zone appeared at all. It proved that SLM loaded with nystatin-alginate microparticles can also induce a satisfactory antifungal effect [81].

The abovementioned studies on the antifungal activity of the nystatin-loaded SLMs described in vitro experiments, but only a few in vivo studies. Geerts et al. investigated the in vivo effect of the Visco-GEL TC (Table 1) loaded with 500,000 IU of nystatin on the *C. albicans* growth. They showed that the nystatin-loaded material inhibited fungal growth incompletely. The average log CFU/mL values decreased from 5.79 to 3.79 during 14 days, corresponding to a drop of two orders of magnitude. In addition, a maximum effectiveness of the nystatin-loaded Visco-GEL, which was manifested by the lowest log CFU/mL value of 3.20, was observed on the seventh day [73].

Kumar et al. conducted an in vivo survey on a group of 40 denture wearers to evaluate the antifungal activity of a tissue conditioner (not specified) loaded with nystatin 500,000 IU. They observed that the number of *C. albicans* colonies on the nystatin-loaded SLM decreased from 391 to 43, corresponding to an 89% decrease. This confirmed that SLM loaded with 500,000 IU of nystatin can show effective antifungal action against *C. albicans* [130].

Ibraheem et al. conducted another in vivo study to test the antifungal properties of Visco-GEL (TC, Table 1) loaded with 1,000,000 IU of nystatin against *C. albicans*. The research involved 15 adult males wearing dentures for 2 months. Despite doubling the concentration of nystatin in the SLM, concerning the study of Geerts et al. [73], Ibraheem et al. did not find statistically significant differences in log CFU/mL values. They were 2.33 and 2.91 for the neat and nystatin-loaded Visco-GEL, respectively. They attributed the obtained result to the specific properties of the saliva of the patients taking part in the study, which could cause a decrease in the adhesion of fungi on the surface of the dentures covered with the SLM [131].

#### 4.1.2. Amphotericin B

Amphotericin B could be an alternative to nystatin. Thomas et al. investigated SLMs modified with this polyene antibiotic. They introduced 10 and 20 mg of amphotericin B into the Visco-GEL TC (Table 1) and examined its fungicidal effectiveness against three fungal strains: *C. albicans*, *C. tropicalis*, and *C. krusei*. Visco-GEL loaded with amphotericin B showed a weak antifungal effect, which was surprising because amphotericin B has fungicidal properties [132]. The highest antifungal activity was observed for *C. albicans* on the third day of the study, and it was 27%. Fungicidal effectiveness against *C. tropicalis* and *C. krusei* was lower but longer-lasting. It was 17 and 13% on the third day and 12 and 14% on the sixth day, respectively [71].

Bassi et al. compared the influence of amphotericin B and nystatin on the properties of *C. albicans* biofilms formed on the silicone Silagum-Comfort Soft Relining LT-SLM (Table 3). Amphotericin B inhibited the growth of only 50% fungal colonies, whereas nystatin completely inhibited *C. albicans* growth at the MIC of 15.63 mg/mL. The antifungal activity of the nystatin-loaded SLM decreased with time, and it lasted on a satisfactory level for 8 h [93].

### 4.2. Azole Drugs

The modifications of SLM with azole drugs have also been extensively studied.

#### 4.2.1. Fluconazole

Falah-Tafti et al. investigated in vitro the antifungal effect of the Acropars TC (Table 1) enriched with fluconazole 1, 3, 5, and 10 wt.%. They found that the higher the fluconazole concentration, the higher the antifungal activity against *C. albicans*. Compared to the neat material, fluconazole 1 wt.% was insufficient to produce a difference in the CFU/mL, whereas 3 and 5 wt.% decreased the CFU/mL. However, only the introduction of 10 wt.% of this triazole resulted in a complete inhibition of fungal growth. They concluded that 10 wt.% of fluconazole was an effective concentration to inhibit *C. albicans* growth [78].

Sharma et al. loaded the Visco-GEL TC (Table 1) with 1, 3, 5, and 10 wt.% of fluconazole and investigated the *C. albicans* growth inhibition zone after 24 h and 7 days. A growth inhibition zone did not appear around samples with 1 and 3 wt.% of fluconazole, whereas it appeared around samples with 5 and 10 wt.% of fluconazole and lasted 24 h. Consequently, they concluded that 5 wt.% of fluconazole was the minimum working concentration in SLM, but its effectiveness in inhibiting fungal growth was low [76].

Kumar et al. conducted an in vivo survey to evaluate the antifungal activity against *C. albicans* of a tissue conditioner (not specified) loaded with fluconazole 10 wt.% on 40 denture wearers. They observed that the number of *C. albicans* colonies on the fluconazole-loaded TC samples decreased from 380 to 153, corresponding to a 60% drop [130]. This result implies that the antifungal activity of fluconazole observed in vivo may be weaker than that observed in vitro.

#### 4.2.2. Itraconazole

Chow et al. examined the antifungal effect of itraconazole incorporated into three TCs, Coe-Soft, Visco-GEL, and Fitt (Table 1) in 1, 3, and 5 wt.%. They investigated the *C. albicans* growth inhibition zone for 14 days, both with and without saliva. They observed that the IZD increased with the itraconazole concentration and was larger in saliva, likely due to easier itraconazole diffusion into the aqueous environment [62]. The maximum inhibitory effect on the growth of *C. albicans* was observed at a concentration of itraconazole 5 wt.% in less than 4 days. The IZD ranged from 25.0 to 34.3 mm without saliva and 36.7 to 40.3 mm with saliva. That study also showed that Fitt was less effective in inhibiting fungal growth than Coe-Soft and Visco-GEL [62]. This was attributed to the lower flowability of Fitt compared to Coe-Soft and Visco-GEL [133]. Taking into account the recommended daily dose of itraconazole, which is 200 mg/day [113] (Table 7), itraconazole 5 wt.% did not exceed this value [62].

Bueno et al. investigated antifungal effect of itraconazole 25.5 wt.% (0.256 g of itraconazole per 1 g of SLM powder) on antifungal properties of two TCs, Softone and Trusoft (Table 1). The itraconazole concentration, corresponding to its MIC value of 0.256 g/mL, allowed to inhibit the growth of more than 90% of *C. albicans* colonies, regardless of the incubation time, 1, 2, 7, and 14 days [34]. It showed that a fungicidal effect, lasting at least 14 days, was achieved for Softone and Trusoft loaded with itraconazole. However, the itraconazole content exceeded the recommended daily dosage of this drug (Table 7) [113].

#### 4.2.3. Clotrimazole

Sunil et al. investigated the effect of clotrimazole, an imidazole derivative, on the antifungal properties of the Visco-GEL TC (Table 1) against *C. albicans*. They found that a 100% inhibition of *C. albicans* growth was possible for 14 days, with a maximum drug release observed on the seventh day for material containing 200 mg of clotrimazole [70]. However, this amount of clotrimazole should not be recommended for use in SLM because it exceeds the recommended daily dose of this drug (Table 7, [113]) four times.

Vojdani et al. tested the antifungal effect against *C. albicans* of an unknown silicone SLM loaded with clotrimazole 1 wt.% in long-term water storage for 1, 30, and 60 days. The neat material was colonized with fungi, for which 6.5 × 10^6^, 5.8 × 10^6^, and 6.1 × 10^6^ CFU/mm^2^ were determined after 1, 30, and 60 days, respectively. Clotrimazole only caused a neglecting fungicidal effect, as CFU/mm^2^ have retained the same order of magnitude (2.6 × 10^6^, 3.9 × 10^6^, and 4.6 × 10^6^ CFU/mm^2^ determined after 1, 30 and 60 days, respectively). It proved that 1 wt.% of clotrimazole was insufficient to give the S-SLM antifungal properties [89]. It was in contrast to the result obtained by Pigno et al., who found that a facial prosthetic silicone elastomer loaded with clotrimazole 1 wt.% showed an antifungal effect against *C. albicans* for 5 months in the in vitro experiment. Moreover, they did not observe an intensification of the material antifungal activity and therefore recommended 1 wt.% of clotrimazole as a concentration that could be optimal for clinical use [134].

**Figure 6:**
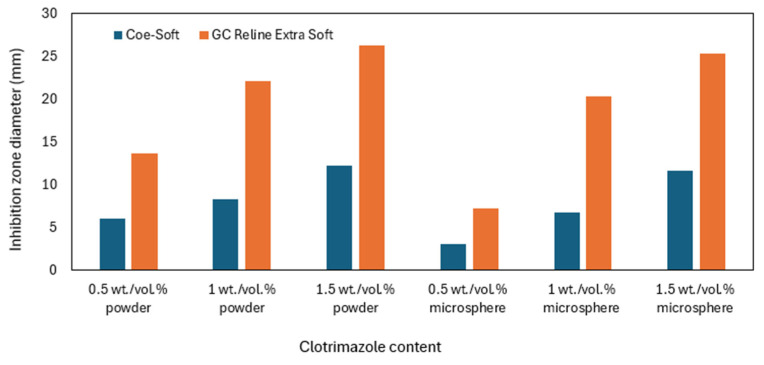
Comparison of the IZD for Coe-Soft and GC Reline Extra Soft SLMs modified with 0.5, 1, and 1.5 wt./vol.% of clotrimazole powder and microspheres. Based on [63].

Pachava et al. also tested the fungicidal effect of clotrimazole-loaded SLMs against *C. albicans*. They modified the Coe-Soft TC (Table 1) and silicone GC Reline Extra Soft LT-SLM (Table 3) with clotrimazole 0.5, 1, and 1.5 wt./vol.% as a powder and microspheres. They showed that the higher the clotrimazole concentration, the larger the growth inhibition zone, indicating increasing antifungal activity. Specifically, they demonstrated that the silicone LT-SLM and clotrimazole powder were more effective in inhibiting fungal growth than the TC and clotrimazole microspheres (Figure 6) [63].

#### 4.2.4. Ketoconazole

Bueno et al. investigated the antifungal effect of 13 wt.% of ketoconazole (0.128 g of ketoconazole per 1 g of SLM powder) on antifungal properties of two TCs, Softone, and Trusoft (Table 1). They demonstrated a 14-day lasting fungicidal effect of both TCs loaded with ketoconazole at the concentration corresponding to its MIC (0.128 g/mL), which was below the recommended daily dosage of this drug (200 mg/day, Table 7, [113]). The growth inhibition of more than 90% of *C. albicans* colonies, regardless of the incubation time (1, 2, 7, and 14 days) was observed [34].

Sunil et al. observed that a 100% inhibition of *C. albicans* growth was possible for 14 days for the Visco-GEL TC (Table 1) containing 200 mg of ketoconazole, and a maximum drug release was between the sixth and seventh day of the study [70]. That amount corresponded to the recommended daily dose of this drug (200 mg, Table 7, [113]).

Barua et al. confirmed the antifungal activity against *C. albicans* of the Visco-GEL TC (Table 1) loaded with 5 and 10 wt.% of ketoconazole. The IZD was 32 and 31 mm, respectively, after 24 h and 7 days. The unchanged IZD indicated a stable ketoconazole release and a 7-day lasting antifungal effect [72].

#### 4.2.5. Miconazole

Radnai et al. demonstrated the antifungal effect of the miconazole-loaded Visco-GEL TC (Table 1) against *C. albicans*. They introduced 5, 10, 15, 20, and 25 vol.% of 24 mg/mL miconazole oral gel into the SLM. They found that the higher the miconazole concentration, the larger the growth inhibition zone and the higher the antifungal activity. The most intense increase in the IZD, by almost 90%, occurred when the miconazole concentration increased from 5 to 10 vol.% (the IZD was 10.29 and 19.47 mm, respectively). A further increase in the miconazole concentration resulted in a smaller increase in the IZD, ranging from 21.16 to 23.39 mm as the miconazole concentration increased from 15 to 25 vol.%. To conclude, 10 vol.% of the 24 mg/mL miconazole oral gel can be regarded as an optimal concentration in the Visco-GEL to give it antifungal properties [77]. Additionally, Radnai et al. found that water immersion decreased the antifungal activity of Visco-GEL modified with 20 vol.% of the miconazole gel. The longer the water immersion time, the lower the IZD, which decreased by 18, 20, and 34% after 24 h, 1 week, and 4 weeks, respectively [77].

Bueno et al. investigated the antifungal effect of miconazole 25.6 wt.% (0.256 g of miconazole per 1 g of SLM powder, corresponding to its MIC value of 0.256 g/mL) on antifungal properties of two TCs, Softone and Trusoft (Table 1). They observed the growth inhibition of more than 90% of *C. albicans* colonies, regardless of the incubation time, which was 1, 2, 7, and 14 days [34]. It showed the stable, 14-day lasting fungicidal effect of the miconazole-loaded TCs. However, the miconazole concentration exceeded the recommended daily dosages of this drug (50mg/day, Table 7, [113]).

Chopde et al. demonstrated that the miconazole presence in Visco-GEL and GC Soft Liner TCs (Table 1) resulted in similar antifungal activity, which was manifested in the approximate IZDs of 8 and 19 mm, respectively, for Visco-GEL and GC Soft Liner [39].

### 4.3. Chlorhexidine Salts

The antifungal properties of SLMs modified with chlorhexidine salts also have been the subject of several studies. Most of these studies have utilized chlorhexidine diacetate [34,64,65,66,67,68,69,72,85], a common disinfectant used in hospitals and public places [135]. Since dental materials containing chlorhexidine diacetate can irritate oral mucosa, and have a noticeable smell of acetic acid and a bitter taste [136], chlorhexidine gluconate is used by choice for oral rinses [136,137]. The potential of chlorhexidine gluconate to produce antifungal effects of SLM was also tested [61,74,77,91]. Only one research paper referred to chlorhexidine hydrochloride, and the results of that study did not reveal any antifungal activity of the SLM [64].

#### 4.3.1. Chlorhexidine Diacetate

Bertolini et al. studied the influence of 0.5, 1, and 2 wt.% of chlorhexidine diacetate and chlorhexidine hydrochloride on the antifungal properties of two TCs, Coe-Soft and Trusoft (Table 1). They found that the chlorhexidine salt was crucial for achieving the antifungal activity of SLM. Any antifungal activity against *C. albicans* was observed for both SLMs modified with chlorhexidine hydrochloride. Conversely, chlorhexidine diacetate was demonstrated to be an effective antifungal compound when added to both SLMs. A fungicidal activity increased with increasing chlorhexidine diacetate content. However, 0.5 wt.% of this salt was insufficient to achieve an antifungal effect, as no growth inhibition zone appeared around the samples. A growth inhibition zone was present at concentrations of 1 and 2 wt.% of chlorhexidine diacetate [64].

Albrecht et al. confirmed that 1 wt.% of chlorhexidine diacetate can be sufficient to trigger antifungal activity against *C. albicans* of SLM, which can last for 4 weeks. Two TCs, Soft Confort and Trusoft (Table 1) were utilized in that study, with Soft Confort exhibiting higher antifungal activity than Trusoft (the IZD values were almost twice as large for Soft Confort compared to Trusoft, 7.82 and 4.01 mm, respectively). The authors suggested that the differences in antifungal activity could be due to variations in the surface porosity of SLMs. They hypothesized that the greater porosity and elasticity of Soft Confort can facilitate the release of chlorhexidine diacetate from its samples [69].

Bertolini et al. showed that Trusoft (TC, Table 1) loaded with chlorhexidine diacetate had a higher antifungal activity against *C. albicans* than Coe-Soft (TC, Table 1). Different strengths of intermolecular interactions between chlorhexidine diacetate and the polymer constituting the SLM powder can be responsible for it. The values of a polymer water contact angle (WCA) of PMMA (Coe-Soft, Table 1) and PEMA (Trusoft, Table 1), which are 64 and 78°, respectively, show that the Trusoft surface is less hydrophilic than that of Coe-Soft [138]. It suggests that chlorhexidine diacetate may have a weaker chemical affinity to PEMA, constituting a powder component of Trusoft. As a result, its leaching from Trusoft can be easier than from Coe-Sof, which can promote antifungal action [64].

Abraham et al. demonstrated that the presence of 0.5, 1.5, 2.5, as well as 3.5 wt.% of chlorhexidine diacetate in the Vertex Soft ST-SLM (Table 2) resulted in its antifungal activity against *C. albicans*, which was manifested by the appearance of a growth inhibition zone. The IZDs depended on the concentration of chlorhexidine diacetate and incubation time in artificial saliva (2 days, 2 weeks, and 4 weeks). The higher the chlorhexidine diacetate concentrations, the larger the IZD. The largest growth inhibition zones were observed after 2 days, followed by a decrease over time, indicating a decreasing release of chlorhexidine salt into the artificial saliva. Specifically, the SLM enriched with chlorhexidine diacetate 3.5 wt.% exhibited the largest IZD after all incubation periods: 18.55 mm (2 days), 17.3 mm (2 weeks), and 15.66 mm (4 weeks). The results also demonstrated that the fungicidal activity lasted 4 weeks [85].

Patel et al. demonstrated that chlorhexidine diacetate of 4.5, 6, 9, and 12 wt.% produced the antifungal effect of a model SLM consisting of PEMA powder and tetrahydrofurfuryl methacrylate liquid. They found that the higher the concentration of chlorhexidine diacetate, the lower the CFU/mL. However, only the samples containing 9 and 12 wt.% chlorhexidine diacetate exhibited an inhibitory effect for 5 h, and only the material loaded with 12 wt. of % chlorhexidine diacetate almost completely inhibited the growth of *C. albicans* [120].

Bueno et al. showed that the chlorhexidine diacetate concentration that was able to inhibit over 90% of *C. albicans* colonies was 6.4 wt.% (0.064 g of chlorhexidine diacetate per 1 g of SLM powder), regardless of the incubation time (24 h, 48 h, 7 days, and 14 days) and the TC used, Softone and Trusoft (Table 1) [34].

#### 4.3.2. Chlorhexidine Gluconate

Radnai et al. demonstrated that incorporating 1 wt./vol.% solution chlorhexidine gluconate at concentrations from 5 to 25 vol.% into the Visco-GEL TC (Table 1) did not produce an antifungal effect against *C. albicans* [77].

AlHamdan et al. treated the acrylic GC SOFT LT-SLM (Table 4) with 0.12 wt.% chlorhexidine digluconate solution. They found that the log10 CFU/mL was 2.36, which suggests some antifungal activity [74].

Krishnaveni et al. showed that immersion of the acrylic GC SOFT LT-SLM (Table 4) in a 2% chlorhexidine solution (salt not specified, but the way of its introduction suggests chlorhexidine gluconate) produced only a negligible antifungal effect against *C. albicans*. The CFU/mL determined for samples immersed in the chlorhexidine solution was slightly lower than CFU/mL for the samples kept in water, but that difference did not have statistical meaning [80].

Rathore et al. determined the growth inhibition zone of the silicone GC Reline Extra Soft LT-SLM (Table 3) enriched with 2 wt./vol.% of chlorhexidine salt (salt not specified, but the way of its introduction suggests chlorhexidine gluconate) after 24 and 72 h. They observed small inhibition zones around samples with IZDs of 5.44 and 6.31 mm, respectively. It indicated a low ability to inhibit *C. albicans* growth [91].

The findings from studies on SLMs modified with chlorhexidine gluconate demonstrated it as ineffective antifungal compound.

## 5. Comparison of Antifungal Compounds by Their Influence on Antifungal Activity of SLMs

Bassi et al. showed that the addition of nystatin into the silicone Silagum-Comfort Soft Relining LT-SLM (Table 3) demonstrated more effective antifungal action in comparison to amphotericin B and fluconazole. Nystatin in a concentration of 15.63 µg/mL and 62.5 µg/mL (depending on the sterility control) caused a complete inhibition of *C. albicans* growth. Amphotericin B and fluconazole caused only partial inhibition. For example, 80% inhibition was observed for 250 µg/mL of amphotericin B and 500 µg/mL of fluconazole [93].

Falah-Tafti et al. confirmed that nystatin can have higher antifungal activity than fluconazole. Nystatin 1 wt.% caused a complete inhibition of *C. albicans* growth of the Acropars TC (Table 1), whereas fluconazole 1 wt.% did not show a difference in the CFU/mL compared to the neat material. A complete inhibition of the fungal growth was possible for fluconazole 10 wt.% [78].

Sunil et al. demonstrated that nystatin can produce a stronger antifungal effect than clotrimazole, and ketoconazole. They observed a complete inhibition of the *C. albicans* growth for the Visco-GEL TC (Table 1) loaded with 500,000 IU, whereas 200 mg of azoles were required to achieve the same effect. Regarding the recommended daily doses of drugs used, only the nystatin content in the SLM was significantly lower and was about 25% of the recommended daily dose. In addition, nystatin produced a 14-day lasting complete inhibition of fungal growth [70].

Bueno et al. compared the fungicidal effects of nystatin, itraconazole, ketoconazole, miconazole, and chlorhexidine diacetate introduced into two TCs, Softone and Trusoft (Table 1). Each drug showed a fungicidal effect against *C. albicans* at minimum inhibitory concentrations (MIC), regardless of the TC. The decreasing order of MIC values: itraconazole = miconazole (0.256 g) > ketoconazole (0.128 g) > chlorhexidine diacetate (0.064 g) > nystatin (0.032 g), demonstrates an increasing antifungal activity, showing nystatin as the strongest antifungal compound. This antibiotic also exhibited the longest-lasting antifungal effect, lasting for 14 days. These findings suggested that the polyene antibiotics’ mechanism inhibiting fungal growth is more effective than those of azole drugs, and chlorohexidine diacetate [34].

Chopde et al. compared the influence of nystatin, fluconazole, and miconazole on the antifungal activity against *C. albicans* of two TCs, Visco-GEL and GC Soft Liner (Table 1) by measuring the IZD [39]. They found that the IZD increased in the following order: nystatin (~10.5 mm) < miconazole (~18.5 mm, which corresponds to a 75% increase compared to nystatin) < fluconazole (~23.5 mm, which corresponds to the 125% increase compared to nystatin), indicating an increase in antifungal activity in this order [39]. It suggested that miconazole could be a more effective in antifungal action than nystatin, contradicting the findings of Bueno et al. [34].

Chow et al. observed the *C. albicans* growth inhibition zone around three TCs, Coe-Soft, Visco-GEL, and Fitt (Table 1), loaded with nystatin, fluconazole, and itraconazole. They found that the IZD depended on the drug as well as SLM. The medicines could be arranged by the increasing IZD as follows: nystatin (8.3 mm) < fluconazole (8.6 mm, which corresponds to the 1% increase compared to nystatin) < itraconazole (22.7 mm, which corresponds to the 58% increase compared to nystatin), indicating an increase in antifungal activity in this order [62]. Thus, Chow et al. confirmed the result of Chopde et al. [39], that fluconazole can produce higher antifungal effect than nystatin. Additionally, the results of Chow et al. demonstrated itraconazole as the most effective azole drug. It agreed with the antifungal activity of azoles themselves, among which itraconazole had the lowest MIC (Table 6).

Chow et al. also showed that TCs modified with azole drugs can be ordered by decreasing the inhibitory effect in the following order: Coe-Soft < Visco-GEL < Fitt. Modifications with nystatin resulted in the opposite relationship (Coe-Soft had the weakest antifungal effect, whereas Fitt had the strongest) [62].

Interestingly, some results obtained by Chow et al. did not depend on the drug and TC: (i) the maximum growth inhibition zone was obtained at a concentration of 5 wt.% for each medicine used, (ii) further increase in a drug concentration in the material caused a slight decrease in the IZD, and the drug release reached a plateau (observed only for nystatin and fluconazole, as itraconazole was used in a maximum amount of 5 wt.%), (iii) the maximum growth inhibition zone was observed between 65 and 98 h, (iv) saliva significantly promoted an increase in the growth inhibition zone [62].

Barua et al. compared the influence of nystatin, ketoconazole, and chlorhexidine diacetate on the Visco-GEL TC (Table 1). They found that both medicines had a similar inhibiting effect on the *C. albicans* growth. They were also more active than the antiseptic. The IZD around the TC samples containing nystatin and ketoconazole was approximately 82% bigger than that for chlorhexidine diacetate (17 mm) [72].

Kumar et al. conducted an in vivo survey to evaluate the antifungal activity against the *C. albicans* of a tissue conditioner (not specified) loaded with nystatin 500,000 IU and fluconazole 10 wt.% on a group of 40 denture wearers. They observed a 60% decrease in the number of *C. albicans* colonies for fluconazole and an 89% decrease for nystatin [130]. It indicated that both drugs effectively inhibited the *C. albicans* growth, with nystatin showing a much stronger antifungal effect, which was in agreement with the findings of Bueno et al. [34].

Schneid et al. compared the antifungal effects of the Lynal TC (Table 1) modified with chlorhexidine (salt not specified), clotrimazole, fluconazole, and nystatin. Chlorhexidine salt and azoles were used in 250, 500, and 1000 mg/sample unit, while nystatin was used in 125, 250, and 500 mg/sample unit. They found that all tested antifungal compounds demonstrated antifungal activity. Chlorhexidine and clotrimazole caused similar antifungal activity, which was lower than that of fluconazole and higher than that of nystatin [43].

The above discussion showed that antifungal activity of SLMs primarily depends on the antifungal compound. Among the drugs and antiseptics discussed, amphotericin B, chlorhexidine gluconate and chlorhexidine hydrochloride displayed negligible antifungal activity of the SLMs or none at all. Among the polyene antibiotics, only nystatin demonstrated a significant antifungal effect, while chlorhexidine diacetate was the only chlorhexidine salt that showed antifungal activity. The comparison of results for nystatin and individual azole drugs do not clearly indicate which of them yield a stronger antifungal activity of SLMs. The counting of fungal colonies showed a stronger antifungal activity of nystatin-loaded SLMs compared to azole drugs, unlike the disc diffusion agar test results, which showed nystatin a weaker antifungal agent. A comparison of azole drugs leads to the conclusion that clotrimazole can cause the strongest antifungal effect of SLMs.

## 6. Antibacterial Activity

*Streptococcus mutans* is the most important bacteria constituting the oral biofilm. Its presence synergistically affects the pathogenesis of *C. albicans* and facilitates the adhesion of *C. albicans* to the denture and mucosa. The metabolism of this bacteria leads to the acidification of the oral environment, which promotes the growth of fungi [139,140,141].

Only a few studies have addressed the antibacterial activity of SLMs modified with drugs and antiseptics.

Barua et al. tested the influence of nystatin, ketoconazole, and chlorhexidine diacetate incorporated into the Visco-GEL TC (Table 1) in 5 and 10 wt.% on the antibacterial activity against *S. mutans*. The agar well diffusion test showed that nystatin and ketoconazole did not cause antibacterial action, as no growth inhibition zone appeared around the samples. In turn, chlorhexidine diacetate resulted in a bactericidal effect of the TC. The antiseptic concentration and water immersion time had a negligible impact on the IZD, which ranged from 24 to 26 mm [72].

Songsang et al. showed that 2% chlorhexidine gluconate solution incorporated in 5 vol/vol % into three TCs, GC Soft Liner, Visco-GEL, and COE COMFORT (Table 1), resulted in antibacterial activity against *S. mutans*. The IZD of 23, 20, and 20 mm appeared respectively around the samples of modified TCs, while it did not appear around the sample of neat TCs [61].

The results indicated that modifying SLMs with nystatin and azole drugs did not enhance their antibacterial activity. This limitation might present a challenge for future research aimed at developing new SLMs that could effectively target both fungi and bacteria. The findings also suggested that simultaneous antifungal and antibacterial activity can be achieved by using chlorhexidine diacetate.

## 7. Biocompatibility

Testing the biocompatibility of SLMs is essential to ensure that they are safe for use in contact with human tissues and cells. However, the literature provides limited information on the biocompatibility of SLMs loaded with medicines and antiseptics.

Hotta et al. conducted an in vivo study to assess the biocompatibility of the Trusoft TC (Table 1) loaded with nystatin, ketoconazole, and chlorhexidine diacetate, respectively in 0.032, 0.128, and 0.064 g/1 g of powder, which corresponded to their MICs against *C. albicans* (taken from [34]) [66]. The modification of Trusoft with nystatin and chlorhexidine diacetate did not negatively affect the rats’ oral tissues for 14 days. Conversely, the ketoconazole-loaded SLM decreased the keratin thickness, keratin area, and thickness of the cellular compartment. The high biocompatibility obtained for nystatin and chlorhexidine diacetate probably resulted from their lower MIC values than that of ketoconazole. The MICs of nystatin and chlorhexidine diacetate were also lower than their recommended daily doses (Table 7) as well as the lethal doses (lethal dose of nystatin and chlorhexidine diacetate in mice is 0.2 g/kg and 2 g/kg, respectively). It is worth noting that the ketoconazole recommended daily dose is 200 mg, which is very close to its lethal dose of 166 mg/kg. Therefore, the use of ketoconazole may produce high risk of side effects, such as liver injuries and adrenal gland problems [66].

Songsang et al. tested the impact of 30 vol./vol.% of nystatin oral suspension and 5 vol./vol.% of 2 wt.% chlorhexidine gluconate mouthwash incorporated into three TCs, GC Soft Liner, Visco-GEL, and COE COMFORT (Table 1) on a human gingival fibroblast cell lines. They did not detect cell cytotoxicity for the modified materials [61]. However, other studies showed that chlorhexidine salt and nystatin may contribute to unfavorable cell viability. Liu et al. reported that the survival rate of human fibroblasts, myoblasts, and osteoblasts when exposed to more than 0.02% chlorhexidine gluconate solution was lower than 6% [142]. Zheng et al. showed that nystatin was cytotoxic in hamster buccal epithelial cells, which were exposed to 100–200 µg/mL (0.01–0.02%) of nystatin solution and had a lower survival rate. Conversely, cells exposed to nystatin suspension had a higher survival rate, probably due to nystatin insolubility in water [143].

Limited research on the cytotoxicity of SLMs modified with drugs and antiseptics exhibiting antifungal activity highlights the necessity for additional studies in this area. Currently, it can only be suggested that azole drugs may have more significant side effects compared to nystatin and chlorhexidine diacetate. However, this conclusion was primarily based on studies involving ketoconazole.

## 8. Drug Release

Incorporating an antifungal compound into SLM transforms it into a drug delivery system [144].

When characterizing the antifungal activity of drug-loaded SLMs, one typically considers the time of its peak intensity and its total duration. Different materials may vary in the kinetics of antifungal drug release, which depends on the water solubility of a drug and the chemical affinity between polymer, constituting powder component of SLM, and water. Hence, each antifungal drug release pattern may differ for the specific drug and SLM combination [64,145]. Several works focused on this issue for nystatin [70,81] and chlorhexidine diacetate [64,69,85,120].

Sunil et al. showed that the release rate of nystatin, introduced in 500,000 IU, from the Visco-GEL TC (Table 1) reached the peak intensity on the seventh day [70].

Hyun-Jin et al. investigated the in vitro release profile of nystatin enclosed in alginate microparticles from the Dura Conditioner TC (Table 1) over 2 weeks. They observed that 56% of nystatin was released gradually from the SLM loaded with microparticles during the first 2 days. They also found that about 20% of nystatin was not released from the SLM at all. They attributed this nystatin release pattern to isolating nystatin molecules in microparticles [81].

Bertolini et al. studied the influence of chlorhexidine diacetate release, added in 0.5, 1, and 2 wt.% into two TCs, Coe-Soft and Trusoft (Table 1), after water storage at 37 °C for 48 h. They observed that the higher the concentration of chlorhexidine salt, the higher its concentration in the solution. They also noticed that the chlorhexidine diacetate release profile slightly depended on the TC. More antiseptic was released from Trusoft, which was based on PEMA, than from Coe-Soft, which was based on PMMA. It was in agreement with the findings from antifungal activity tests, which showed that Trusoft (TC, Table 1) exhibited a stronger antifungal effect against *C. albicans* than Coe-Soft. As demonstrated earlier, PEMA is more hydrophobic than PMMA, suggesting that chlorhexidine diacetate may have a weaker chemical affinity to PEMA. Consequently, its leaching from Trusoft may be easier than from Coe-Soft, enhancing its antifungal properties [64].

Abraham et al. showed that the release of chlorhexidine diacetate incorporated in 0.5, 1.5, 2.5, and 3.5 wt.% into the acrylic Vertex Soft ST-SLM (Table 2) depended on concentration and incubation time in artificial saliva. They found that the higher the concentration of chlorhexidine diacetate, the more it was washed out from the SLM. Additionally, the longer the incubation time, the less chlorhexidine diacetate was washed out from the SLM. Finally, the material with 3.5 wt.% of chlorhexidine diacetate was the most effective against *C. albicans* and had a peak intensity after the second day [85]. Abraham et al. also suggested that the drug release kinetics include two phases: (i) a rapid surface release phase followed by (ii) a slower diffusion release phase [85]. This observation was similar to the findings of Albrecht et al. for the Soft Confort and Trusoft TCs (Table 1) loaded with 1 wt.% of chlorhexidine diacetate [69]. Albrecht et al. explained that particles of antifungal compounds initially release faster due to proximity to the outside surface. Later, they have to diffuse from the material inside, resulting in a slower release. They also hypothesized that the porosity and elasticity of an SLM can influence a release rate. The easier release of chlorhexidine diacetate from Soft Confort was probably due to its higher porosity and elasticity in comparison to Trusoft [69].

Patel et al. investigated the release of chlorhexidine diacetate from the model SLM (PEMA/tetrahydrofurfuryl methacrylate) loaded with 4.5, 6, 9, and 12 wt.% chlorhexidine diacetate for 14 days. They found that during the first 24 h, the so-called rapid phase occurred, during which 50 to 80% of the total chlorhexidine diacetate amount released migrated from the SLM. In addition, during the first 24 h, chlorhexidine diacetate release was characterized by a constant increase. After 24 h, the release rate of chlorhexidine diacetate decreased significantly. Finally, 6 to 12% of the incorporated chlorhexidine diacetate was released over 14 days [120].

To summarize, the release profiles of the antifungal compounds depended on their type and concentration in SLMs, as well as the incubation time and environment (aqueous or nonaqueous) of the SLMs. However, each compound was released with a maximum peak, which occurred between 24 h and 7 days. This is a wide time frame that would require more explanation. On the other hand, the occurrence of a release peak after 7 days at most indicates the need for further work on the modification of SLM towards obtaining a more long-lasting antifungal activity.

## 9. Water Sorption

The release of antifungal compounds from SLMs depends on the ethanol content, polymer affinity to water, and the antifungal compound solubility in water and ethanol [146]. Ethanol is commonly added to many SLMs in 6 to 40 vol.%. It works as a plasticizer, solvent, and agent for controlling the amount and rate of a drug release. The solubility of an antifungal compound in ethanol and water promotes its elution from the SLM into the saliva [43].

Lima et al. investigated the effect of nystatin, chlorhexidine diacetate, and ketoconazole added in their MICs, respectively 0.032, 0.064, 0.128 per 1 g of material powder, into two TCs, Softone and Trusoft (Table 1), on water sorption and solubility. They demonstrated that, after 14 days, antifungal compounds usually did not significantly affect the water sorption of both TCs, except chlorhexidine diacetate added to Softone, whose water sorption increased by 98% (the water sorption of the neat Softone and Trusoft was 244.0 and 274.9 µg/mm^3^, respectively). The water solubility of both TCs did not change due to the addition of nystatin (the mean water solubility of both neat TCs was 22.5 µg/mm^3^). Ketoconazole and chlorhexidine diacetate caused increases in water solubility by 37, and 23%, respectively [146].

Patel et al. investigated the effect of 4.5, 6, 9, and 12 wt.% chlorhexidine diacetate on the water sorption of the model PEMA/tetrahydrofurfuryl methacrylate SLM. They found that chlorhexidine diacetate increased the water sorption of SLM. The higher the chlorhexidine diacetate concentration, the higher the water sorption. They also observed that the water sorption increased with the time of immersion in water [120].

The results obtained for the water sorption of SLMs showed that the introduction of chlorhexidine diacetate caused its increase. This phenomenon can be explained by the strongly hydrophilic structure of the chlorhexidine salts (Figure 4). Polyene antibiotics (Figure 2) and azole drugs (Figure 3) have a more hydrophobic structure, which is probably why they did not increase the SLMs’ water sorption. There are no studies in the literature that would present such an approach to the analysis of the water behavior of SLMs modified with various drugs and antiseptics.

## 10. Bond Strength

The modifications of SLMs by introducing antifungal compounds usually result in satisfactory antifungal effects. However, the mechanical properties of the SLMs often deteriorate. It is inconsistent with the general rule that the additives should not worsen the functional properties of the modified material.

One of the main issues with using SLMs is their debonding from the denture surfaces [147]. It creates a space with favorable conditions for biofilm formation, including *C. albicans* colonization [148]. Each SLM possessing antifungal properties becomes ineffective once the bond strength between the SLM and the prosthesis base is weak. It depends on the chemical composition of the prosthesis and SLM, which rules the character and strength of physical interactions between them [149]. For a minimum SLM thickness of 2 to 3 mm, the clinically acceptable bond strength is 0.44 MPa (N/mm^2^) [150].

The bond strength of SLMs modified with medicines and antiseptics was subjected to several studies [65,69,85].

Sánchez-Aliaga et al. investigated the influence of nystatin, miconazole, ketoconazole, itraconazole, and chlorhexidine salt (not specified) on the peel bond strength of two TCs, Softone and Trusoft (Table 1) to the denture base after 1, 7, and 14 days. They found that peel bond strength varied due to the antifungal compounds and SLM. The peel bond strength of Softone increased for miconazole and decreased for itraconazole. Nystatin, ketoconazole, and chlorhexidine salt did not affect the peel bond strength of Softone. The peel bond strength of Trusoft decreased for itraconazole and chlorhexidine salt. Nystatin, ketoconazole, and miconazole did not affect the Trusoft peel bond strength. It is worth noting that itraconazole worsened the peel bond strength of both materials. When comparing the TCs, it was seen that Softone loaded with nystatin and chlorhexidine salt had significantly higher peel bond strength than Trusoft. Another finding showed that the time of immersion in water usually did not deteriorate the peel bond strength. Specifically, its lowest values were observed after 24 h. Then, they increased within 7 days and remained unchanged until the end of the experiment. Exceptionally, the itraconazole caused a significant decrease in the peel bond strength of both SLMs after 7 and 14 days of immersion in water [65].

**Figure 7:**
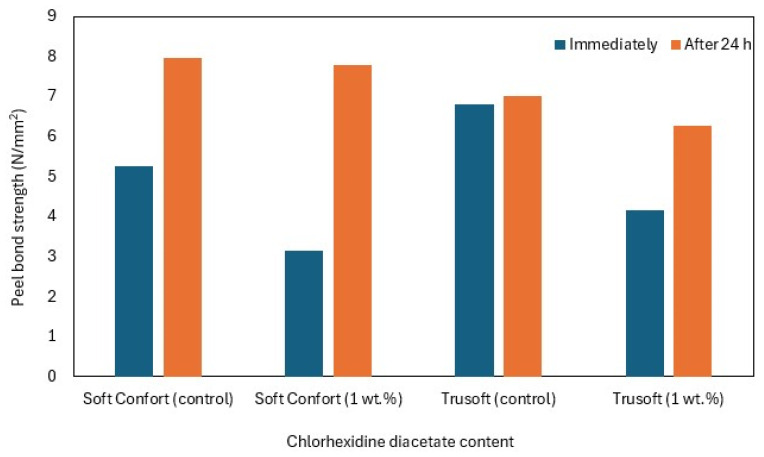
Comparison of the peel bond strength of Soft Confort and Trusoft SLMs modified with 1 wt.% of chlorhexidine diacetate after two measurement periods. Based on [69].

Albrecht et al. investigated the influence of chlorhexidine diacetate 1 wt.% on the peel bond strength of the Soft Confort and Trusoft TCs (Table 1) immediately and after 24 h of immersion in water. Chlorhexidine diacetate caused a decrease in the peel bond strength in all cases. These differences were usually statistically significant, except for Soft Confort after 24 h of immersion in water, which was statistically insignificant (Figure 7) [69].

**Figure 8:**
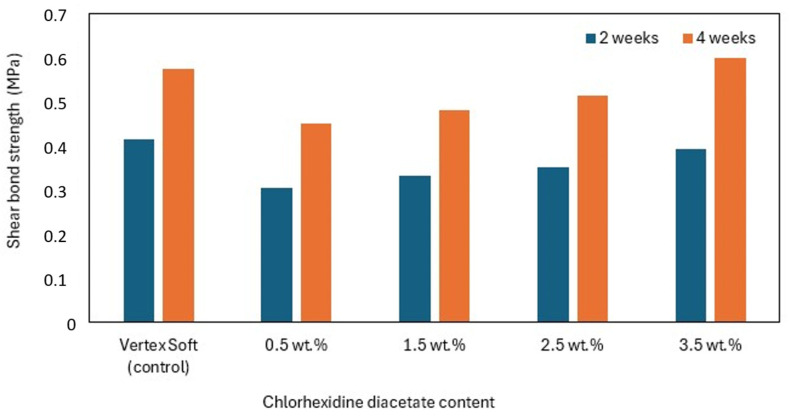
The shear bond strength for Vertex Soft SLM modified with various content of chlorhexidine diacetate after two measurement periods. Based on [85].

Abraham et al. showed that chlorhexidine diacetate introduced into the acrylic Vertex Soft ST-SLM (Table 2) in 0.5, 1.5, 2.5, and 3.5 wt.% generally decreased the shear bond strength. However, the higher the chlorhexidine diacetate concentration, the higher the shear bond strength, which means that the highest reduction in the shear bond strength was observed for chlorhexidine diacetate 0.5 wt.%. It decreased by 27 and 22%, respectively, after 2 and 4 weeks of incubation in artificial saliva. The shear bond strength for chlorhexidine diacetate 3.5 wt.% approached that of the neat Vertex Soft (the neat Vertex Soft had a shear bond strength of 0.415 and 0.574 MPa, respectively, after 2 and 4 weeks of incubation). The authors attributed the initial decreases in the shear bond strength to the increase in the material porosity caused by the presence of chlorhexidine diacetate. The increases in the shear bond strength, observed for higher chlorhexidine diacetate concentrations, probably resulted from the plasticizer leaching, which caused the subsequent increase in stiffness (Figure 8) [85].

The analysis of the bond strength results indicated that each couple’s antifungal compound-SLM can exhibit a unique behavior. It can increase, decrease or remain unchanged. This topic is worth a deeper investigation to explain how the chemical composition of SLMs and the chemical structure of antifungal compounds affect the bond strength between the SLM and denture base. There is a lack of this type of research in the literature.

## 11. Tensile Strength

Tensile strength is a key mechanical property of SLMs that determines their effective response to mechanical stress [151,152]. Two studies investigated this property for SLMs modified with antifungal compounds for clinical use [67,81].

Neppelenbroek et al. investigated tensile strength and percentage of elongation of two TCs, Softone and Trusoft (Table 1) loaded with nystatin, chlorhexidine diacetate, ketoconazole, miconazole, and itraconazole in their MICs, respectively 0.032, 0.064, 0.128, 0.256, and 0.256 g per 1 g of material powder, after 1, 7, and 14 days of water immersion. Miconazole and itraconazole caused a significant reduction in the tensile strength of both SLMs after each immersion time (the tensile strength of the neat Softone was 0.124, 0.131 and 0.167 MPa, and that of Trusoft was 0.146, 0.122, and 0.129 MPa, respectively, after 1, 7 and 14 days). After 14 days, miconazole decreased the tensile strength of Softone by 56% and that of Trusoft by 40%, whereas itraconazole decreased the tensile strength of Softone by 59% and that of Trusoft by 34%. Other drugs did not affect the tensile strength of SLMs. In addition, miconazole the most radically reduced the percentage of elongation of both SLMs after each time of water immersion (the percentage of elongation of neat Softone was 262.9, 266.3, and 364.3%, and that of Trusoft was 331.7, 370.5, and 434.3%, respectively after 1, 7, and 14 days). An average decrease in the percentage of elongation was 159% for this drug [67].

Hyun-Jin et al. investigated the tensile strength of the Dura Conditioner TC (Table 1) modified with nystatin-alginate microparticles. They did not observe a negative effect on the tensile strength. They concluded that nystatin enclosed in alginate microparticles did not interfere with the gelation process, and their presence was neutral for the TC mechanical strength [81].

The results of tensile strength tests indicated that it may decrease due to the introduction of antifungal compounds into SLMs. This is in disagreement with the general principle of material modification, which states that the properties of the materials should not deteriorate. Therefore, more extensive studies should be conducted on the influence of antifungal compounds on the mechanical properties of SLMs. These studies should not be limited to tensile strength; they should also investigate compression and bending strengths as well as modulus of elasticity.

## 12. Hardness

Hardness is a crucial mechanical property of SLMs. It depends on the type and content of low molecular weight substances, such as ethanol and plasticizers, added to SLMs. The greater their content, the lower the initial hardness of the material. In this context, the main drawback of SLMs is the leaching of a plasticizer and solvent that leads to the gradual hardening and loss of functional properties of SLM. A change in hardness acceptable for the clinical use of these materials, typically expressed in the Shore A scale, can range from 13 to 49 in 24 h [153]. In Table 9, the Shore A hardness values of selected SLMs are presented.

Similarly to plasticizers and ethanol, other substances, including antifungal compounds, may also negatively affect the SLMs’ hardness [59,61,68,92]. This issue was discussed in several publications [61,64,68,79,85,92].

Bueno et al. evaluated the Shore A hardness of two TCs, Softone and Trusoft (Table 1), loaded with nystatin, ketoconazole, miconazole, itraconazole, and chlorhexidine diacetate in the amounts corresponding to their MICs, respectively 0.032, 0.128, 0.256, 0.256, and 0.064 g/1 g of the powder. They found that the hardness mainly changed with the TC and water immersion time, while the influence of antifungal compounds was lower. The hardness of the neat Softone increased after 7 days and then decreased after 14 days. The hardness of the neat Trusoft increased after 7 and 14 days of immersion in water. TCs modified with antifungal compounds demonstrated a similar pattern of changes in hardness with a few exceptions. After 24 h of water immersion, the hardness of both TCs decreased for ketoconazole and itraconazole. However, after 7 days of water immersion, the hardness of TCs containing ketoconazole approached that of the unmodified materials. The hardness of the itraconazole-loaded TCs was still lower than that of the neat TCs after 7 and 14 days of immersion in water. The hardness decreased by 50 and 45% for Softone and Trusoft, respectively, after 14 days. The ketoconazole-loaded Trusoft showed a decrease in hardness after 14 days of immersion in water. Other antifungal compounds did not significantly affect the hardness of Softone after 14 days of immersion in water, except for Softone loaded with miconazole and chlorhexidine diacetate, for which the hardness increased after 14 days. On the other hand, all antifungal compounds caused a decrease in the Trusoft hardness after 14 days of immersion in water. The increases in hardness could be explained by plasticizer leaching, whereas the decreases probably resulted from polymer plasticization with water molecules [68].

Kumpanich et al. demonstrated that 20 vol./vol.% of nystatin oral suspension (100,000 IU/mL) added into the GC Soft Liner TC (Table 1) did not affect the A0 hardness. The authors hypothesized that differences in polarity between the nystatin oral suspension and the PEMA constituting the GC Soft Liner powder prevented the SLM softening [79].

Godil et al. showed that the Shore A hardness of the silicone Mollosil LT-SLM (Table 3) increased due to incubation in water, and the fluconazole introduction to this material did not change hardness significantly. They demonstrated that the Shore A hardness of the fluconazole-loaded SLM ranged from 31 to 34 after 1 and 14 days of incubation in water, respectively [92].

Songsang et al. investigated the effect of 30 vol./vol.% of nystatin oral suspension and 5 vol./vol.% of 2 wt.% chlorhexidine gluconate mouthwash incorporated in three TCs, GC Soft Liner, Visco-GEL, and COE COMFORT (Table 1) on Shore A0 hardness after 2 h and 7 days of immersion in water. The hardness of the neat TCs increased with the time of immersion in water, which is typical behavior. The introduction of nystatin and chlorhexidine gluconate usually resulted in a decrease in hardness after 2 h of immersion in water, respectively by 23% for Visco-GEL, 12.5% for COE COMFORT (only for nystatin), and 5% for GC Soft Liner. Chlorhexidine gluconate did not cause a reduction in the hardness of COE COMFORT. After 7 days of immersion in water, the differences in hardness between the modified and unmodified material usually decreased and ranged from 3 to 5%, except for the nystatin-loaded GC Soft Liner and Visco-GEL, for which these differences corresponded to 12 and 23%, respectively [61].

Bertolini et al. studied the influence of chlorhexidine diacetate on the Shore A hardness of two TCs, Coe-Soft and Trusoft (Table 1). The Shore A hardness increased over time [64]. It is a typical water behavior of SLMs because low molecular weight components of SLMs, such as plasticizers and ethanol, are washed out [157]. Initially, the chlorhexidine diacetate, added in 0.5, 1.0, and 2.0 wt.%, in Coe-Soft (based on PMMA), did not affect the hardness. On the other hand, the hardness of Trusoft (based on PEMA) increased with the addition of chlorhexidine diacetate 1 and 2 wt.%. After 7 days of water storage, the hardness of the modified SLMs approached that of their unmodified counterparts [64].

Abraham et al. showed that chlorhexidine diacetate 0.5, 1.5, 2.5, and 3.5 wt.% added into the acrylic Vertex Soft ST-SLM (Table 2) increased the Shore A hardness. The higher the antiseptic content, the more hardness increased, except the result obtained for chlorhexidine diacetate 3.5 wt.% after 4 weeks of incubation in artificial saliva. Hardness increased by a maximum of 7% and 11%, respectively, after 2 and 4 weeks of incubation (the neat Vertex Soft had a hardness of 78.4 and 75.7, respectively, after 2 and 4 weeks of incubation). The material containing chlorhexidine diacetate 3.5 wt.% had a hardness lower than that with 0.5 wt.% of this antiseptic but still slightly higher than neat Vertex Soft. The authors explained this finding by chlorhexidine diacetate interaction with the plasticizer, reducing its ability to solvate the polymer grains and action as a filler due to its powder form [85].

The results obtained for the hardness of modified SLMs showed that it is difficult to estimate the effect of antifungal compounds on their values. They increased due to water or saliva immersion for both the neat SLMs and SLMs loaded with antifungal agents.

## 13. Surface Properties

Surface roughness is another critical property of SLMs, as it influences interactions between microorganisms and the SLM surface in the initial adhesion stage. The rougher the SLM surface, the greater the affinity to biofilm formation [158]. Pores of rough surfaces can promote an attachment of microorganisms and are protected places for their colonization and food collection when cleaning the denture. Surface roughness depends on the SLM type and time of the water storage. Immersion in an aqueous environment results in ethanol and plasticizer leaching, which increases the surface roughness of the SLM [68,159]. Thus, it can be assumed that the longer the service time of SLM, the rougher its surface, and the higher the probability of biofilm formation.

The surface roughness was studied by a few researchers for various SLMs [68,92,158].

Bueno et al. evaluated the effect of nystatin, ketoconazole, miconazole, itraconazole, and chlorhexidine diacetate, added in their MICs, respectively 0.032, 0.128, 0.256, 0.256, and 0.064 g/1 g of the powder, on the surface roughness of the Softone and Trusoft TCs (Table 1) after 24 h, 7 and 14 days of immersion in water. They demonstrated that both the TC and antifungal compound determined the surface roughness. The surface roughness of the neat Softone successively decreased after 7 and 14 days of immersion in water. The addition of ketoconazole and miconazole did not change this behavior. Nystatin caused a decrease in surface roughness after 7 days and an increase after 14 days. Itraconazole and chlorhexidine diacetate caused a progressive increase in the surface roughness. In the case of the neat Trusoft, the surface roughness first decreased and then increased after 7 and 14 days of immersion in water, respectively. This pattern changed due to the addition of antifungal compounds. After 7 days of water immersion, the surface roughness decreased for nystatin and miconazole, whereas it increased for ketoconazole, itraconazole, and chlorhexidine diacetate. After 14 days of water immersion, further decreases in the surface roughness were observed for nystatin, miconazole, and ketoconazole, whereas it increased for itraconazole and chlorhexidine diacetate [68]. It demonstrated that itraconazole and chlorhexidine diacetate can increase the roughness of the SLM surface, which may be detrimental to its functioning.

Godil et al. showed that the surface roughness of the silicone Mollosil LT-SLM (Table 3) modified with fluconazole increased after 1 and 7 days of incubation in water. After 14 days, the surface roughness of the modified material approached that of the unmodified Mollosil [92].

In summary, the surface roughness changed as a result of immersion in an aqueous environment. An analysis of the available articles did not provide clear information on these changes because they may increase or decrease as a result of adding antifungal compounds into SLMs. A deeper investigation into the relationships between the surface roughness of SLMs and their chemical composition, chemical structure, and concentration of antifungal compounds could help to better understand the specificity of drug release from SLMs.

## 14. Conclusions

Numerous studies have shown that the introduction of polyene antibiotics, azole drugs, and chlorhexidine salts into SLMs can result in their satisfactory antifungal activity against *C. albicans*. However, these experiments were typically conducted in vitro and occasionally in vivo and revealed the need for more in vivo studies. Moreover, the modified SLMs exhibited a short-term antifungal effect due to the rapid leaching of antifungal compounds from SLMs into saliva or water. Therefore, further studies should aim to develop SLMs with more stable and long-lasting antifungal activity.

Another conclusion drawn from this literature study is that the biological, physical, and mechanical properties of the modified SLMs were only minimally tested. Knowing the potential cytotoxicity is crucial for clinical applications, whereas understanding the physical properties is essential for the proper functioning of SLMs. This literature review revealed that (i) certain antifungal drugs might increase the cytotoxicity of SLMs, (ii) the release profiles of antifungal compounds exhibited a maximum peak, (iii) physical properties of the modified SLMs, such as the water sorption, bond strength, and tensile strength often deteriorate, (iv) the SLMs’ hardness increased due to the incubation in saliva and it was less influenced by the presence of antifungal compounds, and (v) the SLMs’ surface roughness changed with the incubation time and either increased or decreased depending on the antifungal compound.

In summary, research on antifungal SLMs requires further studies with collaboration among chemists, material scientists, clinicians, and denture users.

## Figures and Tables

**Table 1:** Modifications of commercial TCs (temporary relines) with antifungal compounds are discussed in the review.

Product (Manufacturer, Country)	Composition (Powder/Liquid Ratio)	Antifungal Compound	Content of Antifungal Compound	Source
COE COMFORT™ (COE, GC America Inc., Alsip, IL, USA)	Powder: poly(ethyl methacrylate)/ Liquid: benzyl benzoate and ethanol (6 g/5 mL)	nystatin	30 vol./vol.%	[61]
		chlorhexidine gluconate	5 vol./vol.% of 2 wt.% mouthwash	
Coe-Soft (COE, GC America Inc., Alsip, IL, USA)	Powder: poly(ethyl methacrylate)/ Liquid: benzyl salicylate and ethanol (11 g/8 mL)	nystatin	1, 3, 5, 7, 9 and 11 wt.%	[62]
		itraconazole		
		fluconazole		
		clotrimazole	0.5, 1 and 1.5 wt./vol.% powder and microspheres	[63]
		chlorhexidine diacetate	0.5, 1 and 2 wt.%	[64]
		chlorhexidine hydrochloride		
Trusoft (Boswoth Company, Skokie, IL, USA)	Powder: poly(ethyl methacrylate)/ Liquid: benzyl butyl phthalate, dibutyl phthalate, and ethanol (9 g/6.8 mL)	nystatin	3.1, 6, 11.3, 20.4 wt.%	[34]
			3.1 wt.% ^1^	[65,66,67,68]
		itraconazole	3.1, 6, 11.3, 20.4 wt.%	[34]
			20.4 wt% ^1^	[65,67,68]
		ketoconazole	3.1, 6, 11.3, 20.4 wt.%	[34]
			11.3 wt.% ^1^	[65,66,67,68]
		miconazole	3.1, 6, 11.3, 20.4 wt.%	[34]
			20.4 wt.% ^1^	[65,67,68]
		chlorhexidine diacetate	3.1, 6, 11.3, 20.4 wt.%	[34]
			0.5, 1 and 2 wt.%	[64]
			6 wt.% ^1^	[65,66,67,68]
			1 wt.%	[69]
		chlorhexidine hydrochloride	0.5, 1 and 2 wt.%	[64]
Softone (Bosworth Company, Skokie, IL, USA)	Powder: poly(ethyl methacrylate)/ Liquid: dibutyl phthalate and ethanol (9 g/6.8 mL)	nystatin	3.1, 6, 11.3, 20.4 wt.%	[34]
			3.1 wt.% ^1^	[65,67,68]
		itraconazole	3.1, 6, 11.3, 20.4 wt.%	[34]
			20.4 wt.% ^1^	[65,67,68]
		ketoconazole	3.1, 6, 11.3, 20.4 wt.%	[34]
			11.3 wt.% ^1^	[65,67,68]
		miconazole	3.1, 6, 11.3, 20.4 wt.%	[34]
			20.4 wt.% ^1^	[65,67,68]
		chlorhexidine diacetate	3.1, 6, 11.3, 20.4 wt.%	[34]
			6 wt.%	[65,67,68]
Visco-GEL (Dentsply DeTrey, Konstanz, Germany)	Powder: poly(ethyl methacrylate)/ Liquid: triethyl citrate and ethanol (3 g/2.2 mL)	nystatin	not specified	[39]
			30 vol./vol.%	[61]
			1, 3, 5, 7, 9 and 11 wt.%	[62]
			200,000, 300,000, 400,000, and 500,000 IU	[70]
			500,000 IU, 1,000,000 IU	[71]
			5 and 10 wt.%	[72]
			500,000 IU	[73]
			1,000,000 IU	[74]
			100,000 IU, 300,000 IU, 500,000 IU, and 1,000,000 IU	[75]
		amphotericin B	10 mg and 20 mg	[71]
		fluconazole	not specified	[39]
			1, 3, 5 and 10 wt.%	[76]
			1, 3, 5, 7, 9 and 11 wt.%	[62]
		itraconazole		
		clotrimazole	200 mg	[70]
		ketoconazole		
			5 and 10 wt.%	[72]
		miconazole	not specified	[39]
			5, 10, 15, 20 and 25 vol.% of 24 mg/mL oral gel	[77]
		chlorhexidine diacetate	5 and 10 wt.%	[72]
		chlorhexidine gluconate	5 vol./vol.% of 2 wt.% mouthwash	[61]
			5, 10, 15, 20 and 25 vol.%	[77]
Fitt (Kerr Corporation, Romulus, MI, USA)	No information about chemical composition. (1.5 g/1 g)	nystatin	1, 3, 5, 7, 9 and 11 wt.%	[62]
		fluconazole		
		itraconazole		
Lynal (The L.D. Caulk Division, Dentsply International INC., Milford, Germany)	Powder: poly(ethyl methacrylate)/ Liquid: organic phthalate plasticizer and ethanol (3 g/2 mL)	nystatin	125, 250, and 500 mg/sample unit	[43]
			100,000 IU, 300,000 IU, 500,000 IU, and 1,000,000 IU	[75]
		fluconazole	250, 500, and 1000 mg/sample unit	[43]
		clotrimazole		
		chlorhexidine (salt not specified)		
Acropars (Marlik Medical Industries Co., Tehran, Iran)	Powder: ethyl methacrylate copolymer/ Liquid: ethanol and plasticizers (No information about component ratio)	nystatin	1, 3, 5, 10 wt.%	[78]
		fluconazole		
GC Soft Liner (GC Corp., Tokyo, Japan)	Powder: poly(methyl methacrylate)/ Liquid: butyl phthalate butyl glycolate and ethanol (2.2 g/1.8 g)	nystatin	not specified	[39]
			30 vol./vol.%	[61]
			20 wt.%	[79]
		fluconazole	not specified	[39]
		miconazole		
		chlorhexidine gluconate	5 vol./vol.% of 2 wt.% mouthwash	[61]
		chlorhexidine (salt not specified)	2 vol.%	[80]
Soft Confort (Dencril, Pirassununga, Brazil)	Powder: poly(ethyl methacrylate)/ Liquid: phthalate ester and ethanol (No information about component ratio)	chlorhexidine diacetate	1 wt.%	[69]
Dura Conditioner (Reliance Dental Manufacturing LLC, Alsip, IL, USA)	Powder: poly(ethyl methacrylate)/ Liquid: dibutyl-n phthalate and ethanol (No information about component ratio)	nystatin	0.1 wt.% nystatin-alginate microparticles (28.6 wt.% of nystatin in microparticles)	[81]

^1^ The content of the antifungal compound corresponded to its MIC value determined by the authors.

**Table 2:** Modifications of commercial ST-SLM with antifungal compounds discussed in the review.

Product (Manufacturer, Country)	Composition (Powder/Liquid Ratio)	Antifungal Compound	Content of Antifungal Compound	Source
Vertex Soft (Vertex Dental, Soesterberg, The Netherlands)	Powder: poly(ethyl methacrylate)/ Liquid: acetyl tributyl citrate, methyl methacrylate (1.2 g/1 mL)	chlorhexidine diacetate	0.5, 1.5, 2.5 and 3.5 wt.%	[85]

**Table 3:** Modifications of commercial LT-SLMs of the S-SLM type with antifungal compounds discussed in the review.

Product (Manufacturer, Country)	Composition (Base/Catalyst Ratio)	Antifungal Compound	Content of Antifungal Compound	Source
Molloplast-B (Detax GmbH & Co., Ettingen, Germany)	Hydroxyl terminated polydimethylsiloxane, methyl triacetoxysilane, dibutyltin dilaurate, poly(methyl methacrylate) (one-component material)	butyl pyridinium chloride	2.5, 5, 10 wt.%	[90]
		octyl pyridinium chloride	0.65, 1.25, 2.5 wt.%	
GC Reline Extra Soft (GC Dental Industrial Corp., Tokyo, Japan)	Silicon dioxide, vinyl dimethyl polysiloxane, hydrogen polysiloksane, and catalysts (1:1 volume ratio)	clotrimazole	0.5, 1 and 1.5 wt./vol.% powder and microspheres	[63]
			1 wt.%	[89]
		chlorhexidine digluconate	2 wt./vol.%	[91]
Mollosil (DETAX GmbH & Co., Ettlingen, Germany)	Polydimethylsiloxane with functional group (1:1 volume ratio)	fluconazole	100 to 1000 µg/mL	[92]
Silagum-Comfort Soft Relining (DMG Chemisch-Pharmazeutische Fabrik GmbH, Hamburg, Germany)	Addition-cured vinyl polysiloxanes, hydrogen polysiloxanes, and platinum catalyst (1:1 volume ratio)	nystatin	7.8, 15.6, 31.3, 62.5, 125, 250 and 500 µg/mL	[93]
		amphotericin B		
		fluconazole		
Ufi Gel P (UG, VOCO GmbH, Cuxhaven, Germany)	Mixture of different polyalkylsiloxanes, fumed silica, and catalysts (1:1 volume ratio)	Not tested		

**Table 4:** Modifications of commercial LT-SLMs of the A-SLM type with antifungal compounds discussed in the review.

Product (Manufacturer, Country)	Composition (Powder/Liquid Ratio)	Antifungal Compound	Content of Antifungal Compound	Source
GC SOFT (GC Corporation, Sydney, Australia)	No information about chemical composition (2.2 g/1.8 mL)	chlorhexidine digluconate	0.12 wt.%	[74]
Super soft^®^ (GC America Inc., Alsip, IL, USA)	Powder: poly(ethyl methacrylate)/ Liquid: dibutyl phthalate, isodecyl methacrylate, and methyl methacrylate (5 g/4 mL)	Not tested		

**Table 5:** MIC values of nystatin and amphotericin B against *C. albicans* determined in various studies.

Polyene Antibiotic	MIC (µg/mL)	Source
nystatin	0.27–8.1	[98]
	1.0–4.0	[99]
	3.1–12.5	[100]
	0.125–2.0	[101]
	0.125–16.0	[97]
	1.5–6.5	[102]
	0.371–0.921	[103]
amphotericin B	0.25–4.0	[99]
	0.04–0.4	[100]
	0.012–0.19	[104]
	0.032–0.5	[101]
	0.125–0.5	[98]
	0.25–1.0	[105]

**Table 6:** MIC values of fluconazole, itraconazole, clotrimazole, ketoconazole, and miconazole against *C. albicans*.

Azole Derivative	MIC (µg/mL)	Source
fluconazole	0.125–0.5	[98]
	0.064–0.75	[104]
	0.25–16	[101]
	0.12–64	[108]
	31.25–62.5	[109]
itraconazole	0.002–0.094	[104]
	0.063–1.0	[101]
	0.03–2.0	[108]
	0.443–0.58	[110]
clotrimazole	0.125–2.0	[105]
	0.03–8	[108]
	2.6	[111]
	0.008–8	[112]
ketoconazole	32–64	[99]
	0.125–1.0	[105]
	0.399–0.558	[110]
miconazole	0.25–64	[99]
	1.0–10	[98]
	0.12–16	[108]
	18	[111]

**Table 7:** Recommended daily doses of the discussed medicines [113].

Medicine	Daily Dosage
nystatin	1,600,000–2,400,000 IU ^1,2^
amphotericin B	0.25–0.3 mg/kg
fluconazole	100–200 mg
itraconazole	200 mg
clotrimazole	50 mg
ketoconazole	200 mg
miconazole	50 mg

^1^ 1 IU/mL of nystatin corresponds to 0.16 μg/mL [81]. ^2^ 1 IU/mL of nystatin corresponds to 0.27 μg/mL [98].

**Table 8:** The MIC values of chlorhexidine dihydrochloride, chlorhexidine diacetate, and chlorhexidine digluconate against *C. albicans*.

Chlorohexidine Salt	MIC (µg/mL)	Source
chlorhexidine dihydrochloride	<0.125–8	[123]
chlorhexidine diacetate	25	[124]
chlorhexidine digluconate	1–16	[125]

**Table 9:** Mean Shore A hardness values of commercial SLMs after water immersion.

Product (Manufacturer, Country)	Type of SLM	Water Immersion Time	Shore a Hardness	Source
COE COMFORT™ (COE, GC America Inc., Alsip, IL, USA)	TC	2 h	16.32	[61]
		7 d	22.20	
Coe-Soft (COE, GC America Inc., Alsip, IL, USA)		Initial	9.2	[64]
		2 d	13.3	
		7 d	19.2	
Trusoft (Boswoth Company, Skokie, IL, USA)		24 h	15.6	[68]
		7 d	22.8	
		14 d	31.4	
Softone (Bosworth Company, Skokie, IL, USA)		24 h	14.6	
		7 d	19.2	
		14 d	17.5	
GC Soft Liner (GC Corp., Tokyo, Japan)		2 h	21.79	[61]
		7 d	25.56	
Visco-GEL (Dentsply DeTrey, Konstanz, Germany)		2 h	37.13	
		7 d	40.20	
Fitt (Kerr Corporation, Romulus, MI, USA)		Initial	12.7	[154]
		7 d	39.1	
		14 d	38.1	
Vertex Soft (Vertex Dental, Soesterberg, The Netherlands)	ST-SLM A-SLM	24 h	31.7	[84]
		7 d	32.5	
		28 d	33.3	
Molloplast-B (Detax GmbH & Co., Ettingen, Germany)	LT-SLM S-SLM	24 h	55.1	[155]
		7 d	54.5	
		28 d	53.5	
GC Reline Extra Soft (GC Dental Industrial Corp., Tokyo, Japan)		24 h	60.0	
		7 d	61.1	
		28 d	62.5	
Mollosil (DETAX GmbH & Co., Ettlingen, Germany)		7 d	21.0	[156]
		1 m	24.6	
		3 m	23.6	
		6 m	28.4	

h—hours, d—days, m—months.

## Data Availability

Not applicable.
